# Moustiques, Distribution et Richesse Spécifique dans Huit Pays D'afrique: Cap-Vert, Mauritanie, Sénégal, Gambie, Mali, Burkina Faso, Niger et Tchad

**DOI:** 10.48327/mtsibulletin.2021.109

**Published:** 2021-05-31

**Authors:** E.H. Ndiaye, A. Ould Mohamed Salem Boukhary, M. Diallo, D. Diallo, R. Labbo, P. Boussès, G. Le Goff, V. Robert

**Affiliations:** 1 Pôle de zoologie médicale, Institut Pasteur de Dakar, B.P. 220, Dakar, Sénégal; 2 Université de Nouakchott Al-Aasriya, Unité de recherche génomes et milieux (jeune équipe associée à l'IRD), Laboratoire environnement, santé et société LE2S, BP 880, Nouakchott, Mauritanie; Aix Marseille Univ., IRD, AP-HM, SSA, VITROME, Marseille, France; 3 Centre de recherche médicale et sanitaire (CERMES), BP 10887, Niamey, Niger; 4 Unité MIVEGEC, Université de Montpellier, IRD, CNRS, Montpellier, France

**Keywords:** Diptera, Culicidae, inventaire, NUTS1, biodiversité, espèce, Cap-Vert, Mauritanie, Sénégal, Gambie, Mali, Burkina Faso, Niger, Tchad, Sahel, Afrique sub-saharienne, Diptera, Culicidae, inventory, NUTS1, Biodiversity, Species, Cape Verde, Mauritania, Senegal, Gambia, Mali, Burkina Faso, Niger, Chad, Sahel, sub-Saharan Africa

## Abstract

Les moustiques (Diptera, Culicidae) forment une famille d'insectes d'une importance considérable en santé publique. Les mentions de leur présence/absence ont été recherchées dans la littérature et auprès de sites internet spécialisés pour huit pays d'Afrique: Cap-Vert, Mauritanie, Sénégal, Gambie, Mali, Burkina Faso, Niger et Tchad. Au total, 216 espèces ont été répertoriées, appartenant à 13 genres culicidiens: *Anopheles* (48 espèces), *Aedeomyia* (2), *Aedes* (62), *Coquillettidia* (6), *Culex* (54), *Culiseta* (1), *Eretmapodites* (7), *Ficalbia* (3), *Lutzia* (1), *Mansonia* (2), *Mimomyia* (7), *Toxorhynchites* (4) et *Uranotaenia* (19). La présence de ces espèces dans la zone d'étude est certaine sauf pour trois espèces dont la présence est douteuse. Cette richesse spécifique représente 6% de la richesse mondiale. Les pays hébergeant la plus grande richesse spécifique sont le Burkina Faso (162 espèces), le Sénégal (143) et le Mali (110); le pays avec la plus faible est le Cap-Vert (11). Cette richesse est moindre au nord en climat hyper-aride et supérieure au sud en climat sub-humide. Le Tchad est le pays le moins bien inventorié. Toutes les espèces sont considérées comme natives, à l'exception de *Aedes* (*Stegomyia* ) *albopictus* (le moustique tigre asiatique) introduit en 2016 au Mali et peut-être *Ae.* (*Ochlerotatus* ) *caspius* introduit en Mauritanie et *Ae.* (*Stg.* ) *aegypti* introduit à Nouakchott en Mauritanie. Cette synthèse des connaissances pourra être utile en matière de lutte antivectorielle, de santé publique, et de recherches à venir.

## Abstract (en)

Mosquitoes (Diptera, Culicidae) form a family of insects of considerable public health importance. Mention of their presence/absence was tackled in the literature and by specialized websites for eight African countries: Cape Verde, Mauritania, Senegal, Gambia, Mali, Burkina Faso, Niger and Chad. In total, 216 species have been recorded belonging to 13 genera: *Anopheles* (48 species), *Aedeomyia* (2), *Aedes* (62), *Coquillettidia* (6), *Culex* (54), *Culiseta* (1), *Eretmapodites* (7), *Ficalbia* (3), *Lutzia* (1), *Mansonia* (2), *Mimomyia* (7), *Toxorhynchites* (4) and *Uranotaenia* (19). The presence of these species in the study area is certain except for three species whose presence is doubtful. This specific richness represents 6% of the world's richness. The countries with the highest specific richness are Burkina Faso (162 species), Senegal (143) and Mali (110); the country with the lowest richness is Cape Verde (11). This richness is lower in the north in hyper-arid climate and higher in the south in sub-humid climate. Chad is the least well inventoried country. All species are considered native, with the exception of *Ae.* (*Stegomyia* ) *albopictus* (the Asian tiger mosquito) introduced in 2016 into Mali and possibly *Ae.* (*Ochlerotatus* ) *caspius* into Mauritania and *Ae.* (*Stg.* ) *aegypti* introduced into Nouakchott, Mauritania. This synthesis of the knowledge may be useful for vector control, public health, and future research.

## Introduction

Les moustiques (Diptera, Culicidae) forment une famille d'insectes d'une importance considérable en santé publique. En Afrique, ils sont vecteurs de nombreux arbovirus, protozoaires et métazoaires, agents de maladies. Les moustiques ont fait l'objet d'intenses recherches dès la fin du 19^e^ et au début du 20^e^ siècle après qu'ils aient été formellement impliqués dans la transmission des agents de la fièvre jaune, du paludisme et de la filariose de Bancroft (voir références in [45]). Ce sont bien sûr les espèces de moustiques piquant l'Homme qui ont été les plus étudiées, mais les espèces présentant peu ou pas de contact avec les humains ont aussi fait l'objet d'études pour leur intérêt vétérinaire ou pour mieux connaître leur écologie.

Des mises à jour successives concernant l'Afrique au sud du Sahara [56] ont été réalisées pour les Anophelinae et constituent autant de documents de référence [55, 56, 77, 84, 94]. Elles ont été associées à de nombreuses études sur les vecteurs de *Plasmodium* permettant d'identifier les vecteurs majeurs [56, 65, 128, 129]. Mais rien de comparable n'existe pour les Culicinae.

La présente étude procède à un relevé critique des données de la littérature et dresse la liste des espèces de moustiques connues de huit pays sahéliens d'Afrique. Elle signale les pays où les inventaires d'espèces souffrent d'insuffisance notoire.

## Matériel et Méthodes

### Zone d'étude

Le Sahel (qui veut dire rivage ou côte en arabe) désigne habituellement une bande d'environ 5500 km d'ouest en est sur 400 à 500 km, dans la portion nord de l'Afrique sub-saharienne entre le domaine saharien désertique et les savanes du domaine soudanien où les pluies sont régulières au sud. Il s'étend de l'Atlantique (îles du Cap-Vert inclues) à la mer Rouge (Soudan et Erythrée), dans la bande des degrés de latitude nord 16 à 20 (selon les zones) au nord et 12 à 15 au sud. Le climat est semi-aride chaud avec une pluviométrie annuelle variant entre 250 et 500 mm [101]. Plus que la pluviométrie, c'est l'alternance entre une courte saison humide estivale et une longue saison sèche (de huit à neuf mois, souvent absolument sèche) qui est essentielle. En saison sèche, les alizés continentaux, tels que l'harmattan, soufflent un air chaud et sec des secteurs nord/nord-est, souvent chargé de fines particules de sable ou de poussières minérales. En saison des pluies, le régime des vents s'inverse, une mousson du sud/sud-ouest apporte les pluies. Certaines années, la mousson n'atteint pas les latitudes les plus septentrionales, d'où des épisodes de sécheresse parfois sur plusieurs années consécutives, typiques du climat saharien.

Il se dégage une forte impression d'homogénéité de la bande sahélienne. Elle résulte de faibles variations du climat, du relief, des sols et de leur utilisation. Les changements sont progressifs au nord entre Sahara et le Sahel et au sud entre le Sahel et le domaine soudanien. La monotonie des paysages d'immenses cuvettes, de quelques bas plateaux et reliefs granitiques peu accidentés, est seulement brisée par la présence d'importants éléments fluvio-lacustres tels que le lac Tchad et de grands fleuves (Sénégal, Niger et son delta intérieur au Mali, Logone-Chari, Nil).

Le Sahel n'est pas un désert physique et humain. Végétation, hommes, animaux et activités d'agriculture et d'élevage se sont développés dans cet environnement à ressources contraintes. La région est classée par l'Organisation des nations unies parmi les plus pauvres et les plus fragiles au monde (Tableau I). L'accroissement de la population humaine y est constant. Par exemple, la population du seul Niger est passée de 3,4 millions en 1960 à 23 millions en 2020, pour une projection à 45 millions en 2040. Les six pays, Burkina Faso (ci-après Burkina), Mali, Mauritanie, Niger, Sénégal et Tchad sont sur une trajectoire qui va porter leur population de 89 millions en 2015 à 240 millions en 2050, puis à 540 millions en 2100 [50].

**Tableau I:** Principales caractéristiques des huit pays sahéliens considérés dans le présent article Main characteristics of the eight Sahelian countries included in the present article

Pays	Surface (km^2^)	Habitants	Indice de développement humain*	Superficie en eau douce **(%)
**Cap-Vert**	4030	538535 (en 2014)	0,642 (en 2015)	<0,01
**Burkina Faso**	274200	20835401 (en 2020)	0,402 (en 2014)	<0,01
**Gambie**	11300	2173999 (en 2020)	0,460 (en 2017)	11,5
**Mauritanie**	1030700	4005475 (en 2020)	0,520 (en 2017)	<0,01
**Mali**	1240190	19553397 (en 2020)	0,427 (en 2017)	1,68
**Niger**	1267000	22772361 (en 2020)	0,377 (en 2018)	<0,01
**Sénégal**	196720	16209125 (en 2019)	0,505 (en 2017)	2,1
**Tchad**	1284000	15477751 (en 2018)	0,340 (en 2012)	1,9

* L'indice de développement humain est un indice statistique composite utilisé pour classer les pays

** source de l'information Wikipedia, accès le 5 mai 2020

**Figure 1:**
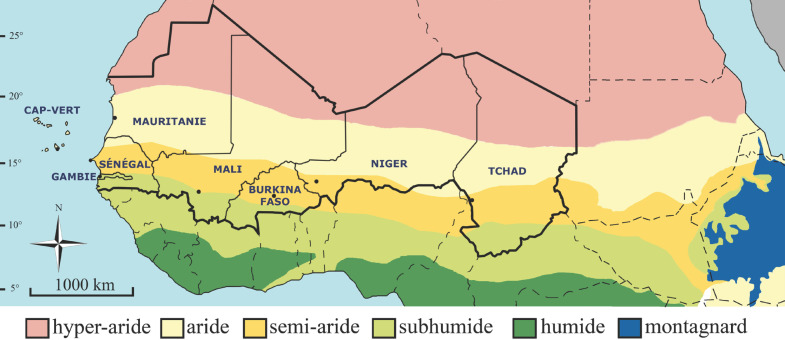
Carte schématique de la frange sub-saharienne de l'Afrique avec mention des huit pays considérés dans le présent article et des zones hyper-aride, aride, semi-aride, sub-humide, humide et montagnarde Schematic map of the sub-Saharan fringe of West Africa identifying the eight countries considered in the present article and the hyper-arid, arid, semi-arid, sub-humid, humid and mountain zones Source FAO, CSAO, OCDE, 2006

La zone d'étude du présent article couvre 8 pays: Cap-Vert, Mauritanie, Sénégal, Gambie, Mali, Burkina, Niger et Tchad (Fig. 1), soit une zone sensiblement différente du Sahel proprement dit. Tous ces pays sont membres de l'organisation intergouvernementale « Comité permanent inter-Etats de lutte contre la sécheresse dans le Sahel (CILSS) » créée le 12 septembre 1973. Ils sont listés parmi les pays les moins avancés sauf le Cap-Vert qui en est sorti en 2007. On notera que les frontières sont restées inchangées depuis l'avènement des indépendances, entre 1960 et 1975 selon les pays. Cinq d'entre eux ont le français comme langue officielle (Burkina, Mali, Niger, Sénégal et Tchad); le portugais et le créole sont les langues officielles au Cap-Vert, l'anglais en Gambie, l'arabe en Mauritanie et aussi au Tchad. Tous sont membres de l'Organisation internationale de la francophonie, ce qui justifie la publication de cet article en français; sauf la Gambie, qui en est observateur. Un pays est insulaire (Cap-Vert) et les autres sont continentaux, avec 3 côtiers (Mauritanie, Sénégal et Gambie). Ces pays ont la totalité de leur territoire situé en zone biogéographique afro-tropicale (=éthiopienne) à l'exception de la Mauritanie, du Mali et du Niger au nord du 20^e^ parallèle et du massif du Tibesti au Tchad, relevant de la zone paléarctique occidentale. En fonction de la latitude, ils relèvent des climats hyper-aride, semi-aride ou sub-humide (Fig. 1). Le climat hyper-aride ou désertique est défini par une pluviosité moyenne annuelle inférieure à 100 mm et aucun mois avec plus de 50 mm en moyenne. Le climat semi-aride ou subdésertique est défini par une pluviosité comprise en 100 et 400 mm, avec un à deux mois recevant plus de 50 mm. Le climat sub-humide, ou tropical semi-aride, est défini par une pluviosité comprise en 400 et 700 mm, avec trois à quatre mois recevant plus de 50 mm [102].

### Moustiques: nomenclature retenue

Seules les espèces culicidiennes valides et dûment répertoriées dans les trois bases de données suivantes sont considérées ici:

- Smithonian Institute, Walter Reed Biosystematics Unit (WRBU) [49, 149];
- Muséum de Londres, Mosquito Taxonomic Inventory (MTI) [108];
- Institut de recherche pour le développement, Montpellier, Arthropodes d'intérêt médical (ARIM) [2].

*Aedes* (*Stegomyia*) *diengi* n'est pas une espèce retenue ici, car mentionnée comme « à décrire », proche de *Ae.* (*Stg.*) *dendrophilus* Edwards, 1921 [26]; mais elle n'a jamais été décrite ensuite. Il en va de même pour trois entités taxonomiques au sein du complexe *Anopheles gambiae*, que certains auteurs ont assigné au rang d'espèces. La première est la forme Bamako, présente dans le sud du Mali et dans le nord de la Guinée [22, 104], la deuxième est la forme Goundry, présente dans le centre du Burkina [28] et la troisième, découverte en 2020, la forme Tengrela, présente dans l'ouest du Burkina [131]. La forme Goundry semble une forme hybride entre *An.* (*Cellia*) *coluzzii* Coetzee & Wilkerson, 2013 et la forme Tengrela qui a été jusqu'alors confondue avec *An. coluzzii*, mais ces deux entités taxonomiques sont génétiquement bien distinctes [131].

La nomenclature retenue est linnéenne et binomiale (conformément au Code international de nomenclature zoologique) à l'exception d'une espèce jumelle d'*Anopheles* (*Cel.*) *rivulorum* Leeson, 1935, dont le nom complet est « l'espèce *Anopheles rivulorum*-like in Cohuet et al 2003 [21] » désignée ci-après *An. rivulorum*-like.

Le nom des espèces est presque toujours concordant entre les trois bases de données. Dans les rares cas de divergence (ordinairement sur une seule lettre), nous avons retenu Harbach [74].

### Moustiques: difficultés taxonomiques

Les principales difficultés taxonomiques rencontrées relèvent des causes suivantes: éclatement d'un taxon en plusieurs, par exemple dans le cas de complexes d'espèces (composés d'espèces morphologiquement semblables) ou de groupes d'espèces (composés d'espèces morphologiquement proches, mais avec des critères diagnostiques au moins à certains stades de développement); élévation du rang de sous-espèce au rang d'espèce; mise en synonymie; révision systématique suivie de changement de genre.

Au niveau des *Anopheles*, le cas du complexe *An. gambiae* (aussi dénommé *An. gambiae s.l.*) est emblématique. On considère aujourd'hui que ce complexe est constitué mais ces deux entités taxonomiques sont génétiquement bien distinctes [131].

La nomenclature retenue est linnéenne et binomiale (conformément au Code international de nomenclature zoologique) à l'exception d'une espèce jumelle d'*Anopheles* (*Cel.*) *rivulorum* Leeson, 1935, dont le nom complet est « l'espèce *Anopheles rivulorum*-like in Cohuet et al 2003 [21] » désignée ci-après *An. rivulorum*-like.

Le nom des espèces est presque toujours concordant entre les trois bases de données. Dans les rares cas de divergence (ordinairement sur une seule lettre), nous avons retenu Harbach [74].

### Moustiques: difficultés taxonomiques

Les principales difficultés taxonomiques rencontrées relèvent des causes suivantes: éclatement d'un taxon en plusieurs, par exemple dans le cas de complexes d'espèces (composés d'espèces morphologiquement semblables) ou de groupes d'espèces (composés d'espèces morphologiquement proches, mais avec des critères diagnostiques au moins à certains stades de développement); élévation du rang de sous-espèce au rang d'espèce; mise en synonymie; révision systématique suivie de changement de genre.

Au niveau des *Anopheles*, le cas du complexe *An. gambiae* (aussi dénommé *An. gambiae s.l.*) est emblématique. On considère aujourd'hui que ce complexe est constitué l'Ouest. Elles peuvent être distinguées avec certitude sur des critères morphologiques au stade larvaire [56] et sur des arguments moléculaires à chaque stade [89].

Dans les années 1960, quatre sous-espèces, ou variétés, d'anophèles ont été érigées au rang d'espèces, *An.* (*Cel.*) *cydippis* De Meillon, 1931, *An.* (*Cel.*) *somalicus* Rivola & Holstein, 1957, *An.* (*Anopheles*) *tenebrosus* Dönitz, 1902 et *An.* (*Ano.*) *ziemanni* Grünberg, 1902.

Au niveau des *Aedes*, le groupe Simpsoni est composé de trois espèces africaines d'*Aedes* du sous-genre *Stegomyia*, dont deux sont présentes en Afrique de l'Ouest, *Ae.* (*Stg.*) *bromeliae* (Theobald, 1911) et *Ae.* (*Stg.*) *lilii* (Theobald, 1910). La troisième, *Ae.* (*Stg.*) *simpsoni* Theobald, 1905 a une distribution limitée à l'Afrique australe [80]. En pratique, les observations antérieures à 1979 ne permettent pas de rapporter une observation à une des espèces du groupe.

Les deux espèces d'*Aedes* du sous-genre *Diceromyia, Ae.* (*Dic.*) *furcifer* (Edwards, 1913) et *Ae.* (*Dic.*) *taylori* Edwards, 1936, ont longtemps été considérées identifiables sur les seuls mâles si bien que la plupart des spécimens collectés étaient attribués à l'ensemble « furcifer-taylori » [71]. Dans les années 1980, leurs identifications sont devenues possibles sur les femelles [47] et sur les larves [76].

Tyson a établi qu'*Aedes* (*Mucidus*) *scatophagoides* (Theobald, 1901) a une distribution strictement asiatique [146]. Pourtant cette espèce, et une espèce proche, *Ae.* (*Muc.*) *sudanensis* (Theobald, 1908), restent largement mentionnées en Afrique de l'Ouest. Dans le présent article, les mentions d'*Ae. scatophagoides* ont, à chaque fois, été assignées à *Ae. sudanensis*.

Au niveau des *Culex, Cx. ethiopicus* Edwards 1912 a été mis en synonymie avec *Cx.* (*Oculeomyia*) *bitaeniorhynchus* Giles, 1901 [73]. En dépit d'une étude moléculaire récente, qui démontre que *Cx. ethiopicus* (au Malawi) et *Cx. bitaeniorhynchus* (en Asie) sont des espèces génétiquement indépendantes avec, comme seule difference morphologique, la forme des écailles de l'aile [103], nous avons conservé la mise en synonymie puisque ces observations moléculaires n'ont pas encore été étayées sur le continent africain.

Le groupe Univittatus comprend au moins trois espèces africaines: *Cx.* (*Culex*) *univittatus* Theobald, 1901, *Cx.* (*Cux.*) *neavei* Theobald, 1906, *Cx.* (*Cux.*) *perexiguus* Theobald, 1903, et peut-être d'autres à décrire [73]. Elles sont morphologiquement très proches (y compris pour les genitalia mâles), si ce n'est indifférenciables, à tous les stades [107]. *Culex neavei* est l'espèce la plus largement distribuée en Afrique au sud du Sahara. *Culex univittatus* est présent dans les milieux d'altitude tempérés d'Afrique australe, mais Harbach a émis des réserves sur sa présence en Afrique de l'Ouest [73]. De sorte que toute identification d'espèces, dans ce groupe, basée uniquement sur des arguments morphologiques, doit être considérée avec précautions.

### Moustiques: origine et critique des données recueillies

Les données utilisées ont été obtenues à partir de quatre sources:

- la littérature scientifique (livres, articles, thèses et rapports);
- les deux sites Internet qui contiennent des indications géographiques de présence par pays (WRBU et ARIM);
- l'expertise des auteurs du présent article;
- et, le cas échéant, l'observation de spécimens de la collection ARIM (voir ci-dessous).

Nous avons utilisé pour la sous-famille Anophelinae, les synthèses de Hamon et al [65], Gillies & de Meillon [56], Gillies & Coetzee [55], Hervy et al [77], Kyalo et al [94] et Irish et al [84]; pour la sous-famille Culicinae, les synthèses de White [148], Knight & Stone [91] et Service [126]; pour le genre *Uranotaenia*, la synthèse de Cunha Ramos [33]; et pour les genres *Mansonia* et *Coquillettidia*, celle de Danilov [35].

Des synthèses nationales relativement récentes pour les Culicidae sont disponibles pour la Mauritanie [106] et le Mali [141].

Pour les autres pays, de telles synthèses n'existent pas et les informations disponibles sont dispersées. Pour chacun des pays de la zone d'étude, les sources bibliographiques additionnelles (par ordre chronologique) ont été les suivantes:

- Cap-Vert: Ribeiro et al [117], Cambournac et al [13, 14], Alves et al [1], Correia et al [23], Salgueiro et al [125];
- Burkina: Hamon & Rickenbach [72], Hamon [62], Hamon et al [67, 71], Balay & Hamon [3], Germain et al [53], Gayral et al [51, 52], Hervy & Couret [75], Huang [80, 82], Robert et al [121, 122, 123];
- Gambie: Findlay & Davey [48], Bertram et al [5], Snow & Boreham [135, 136], Snow [133, 134], Gillies & Wilkes [57], Port & Wilkes [114], Germain et al [54], Bryan et al [11];
- Mali: Bouffard [8], Holstein [78], Hamon [61], Hamon et al [68], Hamon & Brengues [64], Tandina et al [141, 142];
- Mauritanie: Hamon et al [69, 70], Pichon & Ouedraogo [113], Diallo et al [41], Dia et al [36], Sow et al [139], Mint Lekweiry et al [105], Ouldabdallahi Moukah et al [110], Mint Mohamed Lemine et al [106], Ould Lemrabott et al [111];
- Niger: Hamon et al [62, 71], Sales & Ouchoumare [124], Chauvet & Dyemkouma [17], Smith [132], Julvez et al [85, 86], Labbo et al [96, 97, 98, 99, 100], CREC [29, 30, 31, 32], Sous-Comité TIS/HANEA [138], Issa Ado [83];
  Sénégal: Kartman et al [88], Hamon [61], Hamon et al [63, 65, 66, 71], Raymond et al [115], Cornet [24, 25], Cornet et al [26, 27], Camicas et al [15, 16], Diagne et al [37], Huang [82], Hackett et al [60], Traoré-Lamizana et al [145], Diallo et al [38, 39, 40], Biteye et al [6, 7].
- Tchad: Lacan [95], Grjebine [59], Rioux [118, 119], Kerah-Hinzoumbé et al [90], Diarra et al [42, 43].

En pratique, le présent article utilise la nomenclature d'unités territoriales statistiques (NUTS) de l'Union européenne de niveau national, soit NUTS1.

Les notions de densité, ou même simplement d'abondance, n'ont pas été prises en compte, faute de données fiables, sauf exceptions (voir ci-dessous).

L'étude des moustiques n'a pas été conduite avec la même intensité dans les différents pays. Les inventaires d'espèces les plus aboutis ont été effectués à proximité des laboratoires de recherche et dans les zones ayant fait l'objet d'enquêtes répétées (Bobo-Dioulasso au Burkina, Kédougou et Barkedji au Sénégal, Nouakchott en Mauritanie, Niamey au Niger). D'autres régions n'ont pas été prospectées du tout, ou bien l'ont été à une seule saison, ne permettant pas de se faire une idée complète de l'alternance saisonnière des espèces.

À plusieurs reprises, dans le passé, des sous-espèces ont été élevées au rang d'espèces. C'est pourquoi nous avons, autant que possible, conservé à ce stade les informations disponibles sur les sous-espèces. C'est en particulier le cas pour la ssp. *hispaniola* (Theobald, 1903) d'*An.* (*Cel.*) *cinereus* Theobald, 1901, les ssp. nominale et *rupicolus* Lewis, 1937 d'*An.* (*Cel.*) *rhodesiensis* Theobald, 1901, les ssp. nominale et *broussesi* Edwards, 1929 d'*An.* (*Cel.*) *rufipes* (Gough, 1910), les ssp. nominale et *macmahoni* Evans, 1936 d'*An.* (*Cel.*) *sergentii* (Theobald, 1907), la ssp. *mediopunctatus* (Theobald, 1909) d'*Ae.* (*Aedimorphus*) *cumminsii* Theobald, 1903, les ssp. nominale et *arabiensis* (Patton, 1905) d'*Ae.* (*Adm.*) *vexans* (Meigen, 1830), la ssp. *meirai* Ribero, da Cunha Ramos, Capela & Pires, 1980 d'*Ae.* (*Ochlerotatus*) *caspius* (Pallas, 1771), les ssp. nominale et *formosus* (Walker,1848) d'*Ae.* (*Stg.*) *aegypti* (Linnaeus, 1762), les ssp. nominale et *kingii* (Theobald, 1913) de *Cx.* (*Cux.*) *argenteopunctatus* (Ventrillon, 1905), les ssp. nominale et *farakoensis* Hamon, 1955 de *Cx.* (*Cux.*) *grahamii* Theobald, 1910, la ssp. *vicinalis* de Meillon & Lavoipierre, 1944 de *Cx.* (*Cux.*) *invidiosus* Theobald, 1901, la ssp. *nigerrima* Theobald, 1910 de *Mansonia* (*Mansonioides*) *africana* (Theobald, 1901), et enfin la ssp. *conradti* Grünberg, 1907 de *Toxorhynchites* (*Toxorhynchites*) *brevipalpis* Theobald, 1901.

Au contraire, le niveau infra-spécifique n'a pas été pris en compte pour les nombreuses ssp. de *An.* (*Cel.*) *wellcomei* Theobald, 1904 et de *Cx.* (*Cux.*) *pipiens* Linneaus, 1758.

L'examen des sources a été effectué pays par pays, générant ainsi 8 tableaux (Tableaux S1-S8, à consulter sur le site de la Revue).

Le dépouillement de la littérature achevé, une catégorie de distribution a été attribuée à chaque taxon en fonction du pays.

### Moustiques: catégories de distribution

Cinq catégories de distribution, reprises de [120], ont été ici considérées pour chaque taxon:

- taxon présent et autochtone, qui peut se disséminer dans les régions avoisinantes; indiqué ‘Natif'. Un taxon (espèce ou sous-espèce) dont la localité-type se trouve dans le pays est indiqué en souligné ‘Natif';
- taxon considéré comme présent, mais dont la présence n'a pas été formellement démontrée, soit qu'il n'ait pas été observé par défaut d'observations soit que les spécimens n'aient pas été analysés par les méthodes ad hoc, notamment les méthodes moléculaires dans le cas d'espèces jumelles; taxon indiqué ‘Probable';
- taxon présent mais exotique à la zone considérée, parce qu'introduit puis établi (e.g. *Ae. albopictus* au Mali); indiqué ‘Introduit';
- taxon incertain pour la présence, avec des mentions très peu nombreuses et impossibles à vérifier, ou dont la mention fort éloignée de son aire connue de distribution est peu crédible; indiqué ‘Douteux';
- taxon absent, jamais observé, ou ayant fait l'objet d'une mention erronée; indiqué ‘Absent'.

Une éventuelle sixième catégorie pour les espèces à présence ancienne suivie d'une extinction certaine (indiqué ‘Éteinte' in [120]) n'a finalement pas été retenue car la zone d'étude ne présente pas de telles espèces.

Face au constat que le Tchad a été nettement sous-inventorié par rapport aux autres pays du Sahel (voir ci-dessous), nous avons, pour ce pays, extrapolé comme ‘Probable' un taxon ‘Natif' au Burkina et au Niger et présent au Soudan et Soudan du Sud (source WRBU pour ces deux derniers pays).

### Notes sur l'attribution des catégories de distribution

L'attribution de telle ou telle catégorie à certains taxons mérite quelques explications qui sont exposées ci-après par catégorie. Les taxons sont listés par ordre alphabétique des rangs systématiques (genre, sous-genre, espèce et sous-espèce).

‘Natif'

Concernant les Anophelinae, nous confirmons les mentions rapportées pour les 8 pays dans la synthèse la plus récente par Irish et al [84], mais nous la complétons en ajoutant: *An.* (*Cel.*) *argenteolobatus* (Gough, 1910) au Sénégal, *An.* (*Cel.*) *cinctus* (Newstead & Carter, 1910) au Burkina, *An. leesoni* au Niger, *An.* (*Cel.*) *marshallii* (Theobald, 1903) au Burkina et *An.* (*Cel.*) *multicolor* Cambouliu, 1902 en Mauritanie (références ci-dessous).

- *Anopheles* (*Anopheles*) *coustani s.l.* est un complexe de 2 espèces A et B [55], respectivement dénommées *An. coustani* Laveran, 1900 et *An. crypticus* Coetzee, 1994 [19]. Seule la première est présente dans la zone d'étude. La seconde est rapportée uniquement d'Afrique du Sud.
- *Anopheles* (*Anopheles*) *tenebrosus* Dönitz, 1902: espèce présente au Tchad [12] où elle a été mentionnée comme *An. coustani* var. *tenebrosus*, puis élevée au rang d'espèce. Selon Gillies & Coetzee [55], elle n'a pas été retrouvée en Afrique de l'Ouest depuis 1932.
- *Anopheles* (*Cellia*) *argenteolobatus* (Gough, 1910): espèce signalée au Sénégal [149].
- *Anopheles* (*Cellia*) *brucei* Service, 1960: espèce signalée en Gambie [84] à partir d'un spécimen étiqueté Tabana au ‘Natural History Museum, London'. Pas d'autres mentions depuis.
- *Anopheles* (*Cellia*) *brumpti* Hamon & Rickenbach, 1955: espèce décrite au Burkina [72] d'une seule localité (Oué, 43 km nord-ouest de Tougan, Burkina) sur la frontière Burkina-Mali. Pas d'autres mentions depuis.
- *Anopheles* (*Cellia*) *cinctus* (Newstead & Carter, 1910): espèce signalée au Burkina [56, 91, 149] et dans la collection ARIM [2] (sur la base d'une larve étiquetée « Bobo-Dioulasso, 1961 » et rangée dans la boite LAM 435).
- *Anopheles* (*Cellia*) *cinereus* Theobald, 1901: la sous-espèce *An.* (*Cel.*) *cinereus hispaniola* (Theobald) 1903 est mentionnée au Niger [86] et à l'extrême nord du Tchad [118, 119].
- *Anopheles* (*Cellia*) *funestus* Giles, 1900: espèce identifiée par méthode moléculaire au Sénégal (villages proches de Kédougou [92]), au Burkina (villages proches de Ouagadougou [60], au Niger (département de Birni Gaouré, région de Dosso, [83]) et au Tchad (village de Goulmoun [90]).
- *Anopheles* (*Cellia*) *hervyi* Brunhes, Le Goff & Geoffroy, 1999: espèce décrite du sud-est du Niger, région de Guidimouni [10]. Signalée dans la même zone [96]. Endémique du Niger, à distribution très limitée.
- *Anopheles* (*Cellia*) *leesoni* Evans, 1931: espèce signalée lors d'une caractérisation du groupe Funestus par une méthode moléculaire au Niger dans le département de Birni Gaouré, région de Dosso [83] et au Sénégal dans des villages à une vingtaine de kilomètres à l'ouest de Kédougou [92].
- *Anopheles* (*Cellia*) *marshallii* (Theobald, 1903): espèce signalée au Burkina [149] et sur une unique femelle dans la collection ARIM [2] (boite ADU 1055, décembre 1963, Koumbia, Burkina, J. Hamon réc.).
- *Anopheles* (*Cellia*) *multicolor* Cambouliu, 1902: espèce signalée au Niger [85] et en Mauritanie [111]. C'est une espèce paléarctique du Nord Sahara, en limite de distribution dans ces deux pays. Elle a été observée pour la première fois en Mauritanie en 2016 à Nouakchott où sa présence intra-domiciliaire a été suivie jusqu'en 2018. Considérée ici comme ‘Natif' en Mauritanie mais des recherches à venir pourraient amener à revoir ce statut en ‘Introduit' (du Maroc et/ou d'Algérie?).
- *Anopheles* (*Cellia*) *rivulorum* Leeson, 1935: espèce signalée lors d'une caractérisation du groupe Funestus par méthode moléculaire dans des villages du Sénégal à une vingtaine de kilomètres à l'ouest de Kédougou [92] et au Niger (département de Birni Gaouré, région de Dosso) [83].
- *Anopheles* (*Cellia*) *rivulorum*-like (in Cohuet et al 2003): espèce signalée lors d'une caractérisation du groupe Funestus par séquençage de l'ITS2 au Burkina [60] et au Niger (département de Birni Gaouré, région de Dosso) [83]. La large distribution du taxon, également présent au Nord-Cameroun, en Zambie et en Afrique du Sud, incite à penser que d'autres pays de la zone d'étude (en plus du Burkina et du Niger) hébergeraient l'espèce.
- *Anopheles* (*Cellia*) *salbaii* Maffi & Coluzzi, 1958: espèce décrite de Somalie où elle peuple les eaux natronées. Elle a été capturée à plusieurs reprises, et à trois années d'intervalle, vers Zinder au sud du Niger où la majorité des eaux de surface sont plus ou moins natronées [62, 65].
- *Anopheles* (*Cellia*) *theileri* Edwards, 1912: espèce mentionnée au Burkina [51, 65] quoique Gillies & de Meillon estiment que les mentions d'Afrique de l'Ouest se réfèrent probablement à *An.* (*Cel.*) *brohieri* Edwards, 1929 [56].
- *Anopheles* (*Christya*) *implexus* (Theobald, 1903): espèce mentionnée au Burkina, très localisée à une petite forêt relique de palmiers à huile dans la région de Bobo-Dioulasso [51, 62]. Elle n'a plus été mentionnée depuis. Vu la dégradation de cette forêt, il est possible que cette espèce soit, en fait, éteinte.
- *Aedes* (*Aedimorphus*) *vexans* (Meigen, 1830): espèce dont la sous-espèce nominale est mentionnée en Mauritanie en se basant sur les genitalia mâles [70].
- *Aedes* (*Neomelaniconion*) *albothorax* Theobald, 1907: espèce décrite de Gambie, dont l'identification a été confirmée par Zavortink [150].
- *Culex* (*Culex*) *ornatothoracis* Theobald, 1909: espèce présente au Sénégal. Un spécimen mâle dans la collection ARIM [2]
  (boite LAM 507, lame 227, genitalia d'un mâle collecté le 26/09/1953 à Kolda, Sénégal, J. Hamon dét.).
- *Culex* (*Culex*) *sitiens* Wiedemann, 1828: espèce à très large répartition en Asie, Moyen-Orient, Australie, Archipel des Comores, Madagascar, et à répartition plus sporadique en Afrique. Dans la zone d'étude, espèce signalée uniquement au Sénégal, par plusieurs sources, avec des identifications souvent jugées incertaines par leurs auteurs. Les deux sources catégoriques sont [6, 149]. La première ne semble toutefois pas faire la distinction avec d'autres *Culex* morphologiquement proches tels que *Cx.* (*Cux.*) *thalassius* Theobald, 1903.
- *Culex* (*Culex*) *watti* Edwards, 1920: espèce présente au Tchad avec identification morphologique et par MALDI-TOF MS [43].
- *Culex* (*Culiciomyia*) *nebulosus* Theobald, 1901: espèce présente dans tous les pays continentaux de la zone d'étude. La sous-espèce nominale est la seule ici mentionnée.
- *Eretmapodites dracaenae* Edwards, 1916: espèce mentionnée au Burkina, très localisée à une petite forêt relique de palmiers à huile dans la région de Bobo-Dioulasso [51]. Elle n'a plus été mentionnée depuis. Vu la dégradation de cette forêt, il est possible que cette espèce soit, en fait, éteinte.
- *Eretmapodites wansoni* Edwards, 1941: espèce signalée au Sénégal oriental à partir d'un mâle [26]. La sous-espèce *Er. wansoni douceti* Adam & Hamon, 1959 a été décrite puis mentionnée au Burkina [62].
- *Ficalbia uniformis* (Theobald, 1904): espèce connue au Niger par un spécimen en collection ARIM (une femelle montée boite ADU 425. Etiquette: Bassa, cercle de N'Guigmi, Niger, oct 1965, Brunhes réc,).
- *Toxorhynchites* (*Toxorhynchites*) *brevipalpis* Theobald, 1901: la sous-espèce *conradti* Gruenberg, 1907 est mentionnée au Sénégal, Gambie, Mali et Burkina. Il semble que ce soit la seule sous-espèce présente dans de la zone d'étude [116].

#### ‘Probable'

- *Anopheles* (*Cellia*) *flavicosta* Edwards, 1911: espèce signalée en Mauritanie [2, 77] mais la source de l'information reste introuvable. Dans les autres pays continentaux, la présence du taxon est certaine.
- *Anopheles* (*Cellia*) *funestus* Giles, 1900: espèce dont la présence est probable en Mauritanie, Gambie et Mali, c'est-à-dire dans tous les pays continentaux de la zone d'étude où la présence reste non démontrée par méthode moléculaire.
- *Aedes* (*Diceromyia*) *furcifer* (Edwards, 1913): la présence de cette espèce est probable au Mali, au Niger et à Kaédi en Mauritanie [71] car, jusque dans les années 1990, les femelles d'*Ae. furcifer* étaient considérées comme indiscernables de celles d'*Ae. taylori* et traitées comme une seule entité, sous le nom d'*Aedes* du groupe furcifer-taylori.
- *Aedes* (*Diceromyia*) *taylori* Edwards, 1936. La présence de cette espèce est probable en Gambie, au Niger et à Kaédi en Mauritanie [71] avec les mêmes problèmes d'identification précédemment présentés.
- *Aedes* (*Stegomyia*) *bromeliae* (Theobald, 1911): espèce du groupe Simpsoni dont 2 espèces sont présentes dans la zone d'étude: *Ae. bromeliae* et *Ae. lilii*. L'identification morphologique sur la femelle et sur la larve est possible [47, 76], mais délicate et n'a pas toujours été faite. Si bien que la présence d'*Ae. bromeliae*, quoique non-démontrée, est probable en Gambie [48], au Mali [141] et au Niger [71].
- *Aedes* (*Stegomyia*) *lilii* (Theobald, 1910). Pour les mêmes raisons, la présence de cette espèce est probable en Gambie [48], au Mali [141] et au Niger [71].
- *Aedes* (*Stegomyia*) *pseudoafricanus* Chwatt, 1949: espèce suspectée dans le sud-ouest du Sénégal [71] considérée comme probable au Sénégal car (i) c'est une espèce présente en Gambie [136] (ii) cette espèce est typiquement une espèce de mangrove, milieu naturel bien représenté en Casamance.
- *Mansonia* (*Mansonioides*) *africana nigerrima* Theobald 1910: cette sous-espèce (présente au Sénégal, Gambie, Mali, Burkina, Niger et Tchad) devrait aussi être présente en Mauritanie, mais ce n'est pas documenté.

#### ‘Introduit'

- *Aedes* (*Ochlerotatus*) *caspius* (Pallas, 1771): espèce observée pour la première fois en Mauritanie à Nouakchott par Mint Lekweiry et al [105]. C'est probablement une espèce introduite, et son établissement à Nouakchott, ou ailleurs en Mauritanie, reste à investiguer.
- *Aedes* (*Stegomyia*) *aegypti* (Linnaeus, 1762): espèce observée pour la première fois en 2014 à Nouakchott [105]. Cette observation est en faveur d'une introduction récente. Morphologiquement, les spécimens observés relevaient de la sous-espèce nominale. Au Sénégal, la forme claire (var. *queenslandensis*) de la sous-espèce nominale a été signalée dans les environs de Thiès, au village de Pallai [63]. Plus récemment, la présence de la forme claire et de la forme sombre a été observée à Niakhar, suggérant des variations morphologiques en fonction de la saison et de la nature des gîtes larvaires, mais sans différence génétique perceptible entre sous-populations à partir de l'étude de microsatellites [112]. Toujours au Sénégal, la sous-espèce *aegypti* était dominante dans le nord-ouest alors que la sous-espèce *formosus* (Walker, 1848) dominait dans le sud-est [140]. Une clarification sur cette question des sous-espèces morphologiques d'*Ae. aegypti* a été apportée par des études portant sur des échantillons familiaux (provenant d'oeufs issus de couples monosubspécifiques *Ae. ae. aegypti* ou *Ae. ae. formosus*) sur plusieurs populations d'*Ae. aegypti* du Sénégal. La plupart des pontes d'une seule femelle étaient hétérogènes, générant des adultes morphologiquement *Ae. ae. aegypti* aussi bien que *Ae. ae. formosus*. Si bien que la division d'*Ae. aegypti* en deux sous-espèces morphologiques est invalide au Sénégal [44]. Cette conclusion est en plein accord avec des observations similaires en Afrique du Sud [87]. En revanche, des analyses génomiques démontrent clairement l'existence de deux sous-espèces distinctes, la sous-espèce *formosus* étant partout dominante en Afrique et la sous-espèce nominale dans le reste du monde [58, 93, 137].
- *Aedes* (*Stegomyia*) *albopictus* (Skuse, 1895): espèce observée pour la première fois en 2016 dans deux zones (Mopti et Bamako le long du fleuve Niger) au Mali [109]. Pas d'autres mentions dans les autres pays étudiés.

#### ‘Douteux'

- *Anopheles* (*Cellia*) *demeilloni* Evans, 1933: espèce signalée en Mauritanie [69] à partir de larves collectées à Kioun Ouniou Iihegat (Hodh occidental) dans un ruisseau issu d'une source permanente, avec beaucoup de végétation et de débris organiques. Hamon et al (1964) soulignent que la présence de cette espèce en Mauritanie est inattendue. Pas d'autres mentions dans la zone d'étude. Il convient de citer ici Hervy et al [77]: « *An. demeilloni* présente sa forme typique en Afrique de l'Est. En Afrique occidentale, centrale et australe, de nombreuses et importantes variations morphologiques ont été signalées, ce qui nous incite à penser que ce taxon regroupe actuellement un complexe d'espèces. Sa révision, incluant les espèces proches comme *An. carteri* et *An. freetownensis*, nous semblerait très utile. »
- *Anopheles* (*Cellia*) *dureni* Edwards, 1938: espèce signalée au Burkina par Irish et al [84] en se basant fautivement sur ARIM et signalée par [149]. Pas d'autres mentions dans la zone d'étude.
- *Anopheles* (*Cellia*) *melas* (Theobald, 1903): alors que l'espèce n'a jamais été rapportée de localité éloignée du littoral atlantique (où elle est présente en Mauritanie, Sénégal et Gambie et se développe dans des gîtes larvaires d'eau saumâtre), plusieurs mentions récentes ont été faites au Niger. Ces mentions ne proviennent pas d'articles scientifiques mais de rapports venant de deux sources. La première est le Centre de recherche entomologique de Cotonou (CREC) qui propose 4 rapports annuels (datés 2016 à 2019) avec l'analyse de 450, 350, 300 et 299 *An. gambiae s.l.* pour les rapports de 2016, 2017, 2018 et 2019, respectivement [29, 30, 31, 32]. Ces moustiques sont testés par «PCR espèce» (rapports 2016 et 2018), «PCR espèce et PCR formes moléculaires» (rapport de 2017) et «PCR SINE200 (Santolamazza et al 2008) et Scott et al (1993)» (rapport de 2019). La présence d'*An. melas* est rapportée de deux localités: Madarounfa dans le département éponyme et la région de Maradi (8 *An. melas* / 50 *An. gambiae s.l.* testés en 2016; 6/75 en 2019) et Tillabéri dans le département et la région éponyme (3/50 en 1996). La seconde source est un rapport non daté (2019?) du Sous-Comité TIS/HANEA intitulé « Projet pilote d'utilisation de la technique de l'insecte stérile (TIS) pour l'éradication des moustiques vecteurs du paludisme au Niger » [138] qui mentionne des études entomologiques menées par le Programme national de lutte contre le paludisme, selon une méthodologie non précisée, sur des *An. gambiae s.l.* collectés dans 14 sites répartis dans tout le Niger. La présence d'*An. melas* est rapportée par l'étude faite en 2013-2014 dans 3 localités: Boboye dans le département de Birni N'Gaouré et la région de Dosso, Balleyara dans le département éponyme et la région de Dosso, et Madaoua dans le département éponyme et la région de Tahoua; et par l'étude faite en 2016 dans la localité de Boboye précisant qu'*An. melas* représente 94,1% des *An. gambiae s.l.* testés. Il ne semble pas y avoir eu de séquençage des produits de PCR. Au vu de ces indications et de sa biologie larvaire, la présence d'*An. melas* au Niger demande confirmation.
- *Aedes* (*Aedimorphus*) *tauffliebi* Rickenbach & Ferrara, 1965. Espèce signalée au Sénégal [149], sans autre mention par ailleurs en Afrique de l'Ouest.

#### ‘Absent'

- *Anopheles* (*Anopheles*) *coustani* Laveran 1900: espèce considérée absente du Niger depuis que Julvez et al [86] ont estimé que probablement seul *An. ziemanni* est présent au Niger, et que les mentions de *An. coustani* antérieures à 1970 ne distinguaient pas *An. coustani* de *An. ziemanni*. À la suite de [94] puis de [84], nous nous rangeons à cet avis et considérons cette espèce absente du Niger.
- *Anopheles* (*Cellia*) *dthali* Patton 1905: espèce mentionnée au Mali, uniquement par Irish et al [84] se basant sur ARIM, ce qui n'a pas été retrouvé dans ARIM. Considérée donc absente du Mali, quoique sa présence y soit possible en zone subdésertique qui est la zone de prédilection de l'espèce depuis le Maroc jusqu'à l'Inde.
- *Anopheles* (*Cellia*) *gambiae* Giles, 1902: espèce signalée en Mauritanie et au Cap-Vert par WRBU en tant que *An. gambiae s.l.*, soit, très probablement *An. arabiensis* (R. Wilkerson com. pers.)
- *Anopheles* (*Cellia*) *longipalpis* (Theobald, 1903): espèce absente du Mali. L'unique mention, faite par Hamon et al (1961), se réfère à *An. longipalpis domicolus* (sous-espèce maintenant érigée au rang d'espèce = *An. domicolus* Edwards 1906).
- *Anopheles* (*Cellia*) *maliensis* Bailly-Choumara & Adam, 1959: espèce d'altitude connue de la localité-type (Mali) au Fouta-Djalon, Guinée. Egalement signalée au Mali par WRBU [149] (repris par [141]), mais il semble que cela résulte d'une confusion entre la localité type et la République du Mali (Wilkerson, com pers), donc considérée comme ‘Absent' au Mali.
- *Anopheles* (*Cellia*) *moucheti* Evans 1925: espèce mentionnée comme erreur probable du Burkina [84]. Effectivement cela a été corrigé dans la base ARIM. Espèce considérée absente de la zone d'étude.
- *Anopheles* (*Cellia*) *rhodesiensis* Theobald, 1901: espèce dont la sous-espèce *rupicolus* Lewis, 1937 a été fautivement rapportée au Niger par Kyalo et al (2017) (en citant Julvez et al 1998).
- *Anopheles* (*Cellia*) *subpictus* Grassi, 1899: espèce absente du Burkina (Irish et al 2020) et de toute la zone d'étude.
- *Anopheles* (*Cellia*) *schwetzi* Evans, 1934: espèce signalée au Mali [149] puis [141] se basant sur WRBU, mais la source de l'information reste introuvable. Pas d'autres mentions dans la zone d'étude. La distribution de l'espèce est limitée à l'Afrique australe et centrale autour des Grands Lacs [56, 77].
- *Aedes* (*Mucidus*) *scatophagoides* (Theobald, 1901): espèce absente d'Afrique. D'après Tyson [146], *Ae. scatophagoides* a une distribution strictement asiatique; tous les moustiques ainsi identifiés en Afrique sont en fait des *Ae.* (*Muc.*) *sudanensis* (Theobald, 1908).
- *Aedes* (*Neomelaniconion*) *lineatopennis* (Ludlow, 1905): espèce dont l'identification en zone afro-tropicale pose problème dans la mesure où Huang a estimé que la distribution de cette espèce est uniquement orientale et que les spécimens d'Afrique de l'Est et du Sud initialement attribués à cette espèce appartiennent en fait à l'espèce morphologiquement proche *Ae.* (*Neo.*) *mcintoshi* Huang, 1985 [81]. Il nous a donc semblé probable que les identifications de *Ae. lineatopennis* au Burkina [3, 51, 62, 75] et au Mali [68], toutes antérieures à 1985, sont erronées. Aussi, dans la collection ARIM, nous avons examiné 12 femelles de la boite ADU362, étiquetées « BAMA, Cercle de Bobo-Dioulasso, Haute Volta, 11-08-54, *Ae.* (*Neo.*) *lineatopennis*, J. HAMON ORSTOM Dét », et 12 femelles de la boite ADU 313, étiquetées « SOROSSARASSO, Cercle de Bobo-Dioulasso, Haute Volta, 12-10-59, *Ae.* (*Neo.*) *lineatopennis*, J. HAMON ORSTOM Dét ». En se basant sur les critères morphologiques de [81], ces 24 femelles ne sont pas des *Ae. lineatopennis*, ni des *Ae.* (*Neo.*) *circumluteolus* (Theobald, 1908), mais bien des *Ae. mcintoshi*. En conséquence, *Ae. lineatopennis* est considéré ‘Absent' du Burkina et du Mali. Et *Ae. mcintoshi* est considéré ‘Natif' du Burkina et ‘Probable' du Mali.
- *Culex* (*Culex*) *ventrilloni* Edwards, 1920: espèce décrite dans la ville d'Antananarivo, Madagascar, considérée endémique de Madagascar, morphologiquement très proche de *Cx.* (*Cux.*) *simpsoni* Theobald, 1905 [143]. L'observation de 5 spécimens dans le nord du Sénégal [7] est peu crédible, une confusion avec *Cx. simpsoni* est beaucoup plus probable. Espèce considérée ici absente du Sénégal et de toute la zone d'étude.
- *Culex* (*Oculeomyia*) *annulioris* Theobald, 1901 a été rapporté du Nord du Tchad par Rioux dans une courte note [118], mais non dans son article paru peu après qui reprend en détails toutes ses observations [119].
- *Culex* (*Oculeomyia*) *ethiopicus* Edwards, 1912 a été mis en synonymie avec *Cx. bitaeniorhynchus* [73].
- *Uranotaenia* (*Uranotaenia*) *neireti* Edwards, 1920 a été mentionné au Sénégal dans un rapport non publié [15]. Mais cette espèce est considérée endémique de Madagascar [34].

## Résultats

Un tableau de synthèse a été construit pour présenter, visuellement, le statut de distribution de chaque espèce dans chaque pays (Tableau II). Deux-cent-seize espèces ont été inventoriées dans au moins un des huit pays étudiés, mais 3 d'entre elles ont été placées dans la catégorie ‘Douteux' si bien que dans les 8 pays étudiés le nombre total d'espèces présentes est de 213.

**Tableau II:** Distribution des 216 espèces culicidiennes par pays du Sahel. Les pays (colonnes) sont listés d'ouest en est, et les espèces (lignes) sont listés par ordre alphabétique des rangs systématiques (sous-famille, genre, sous-genre et espèce). Les catégories ‘Natif', ‘Natif', ‘Probable', ‘Introduit' et *‘*Douteux' sont définies dans les matériels et méthodes. Distribution of the 216 culicidian species per country of the Sahel. The countries (columns) are listed from West to east, and the species (lines) are listed by alphabetic order of systematic ranks (sub-family, genus, sub-genus and species). The categories ‘Natif', ‘Natif', ‘Probable', ‘Introduit' and ‘Douteux' are defined in the section Materials and Methods.

**Figure:** 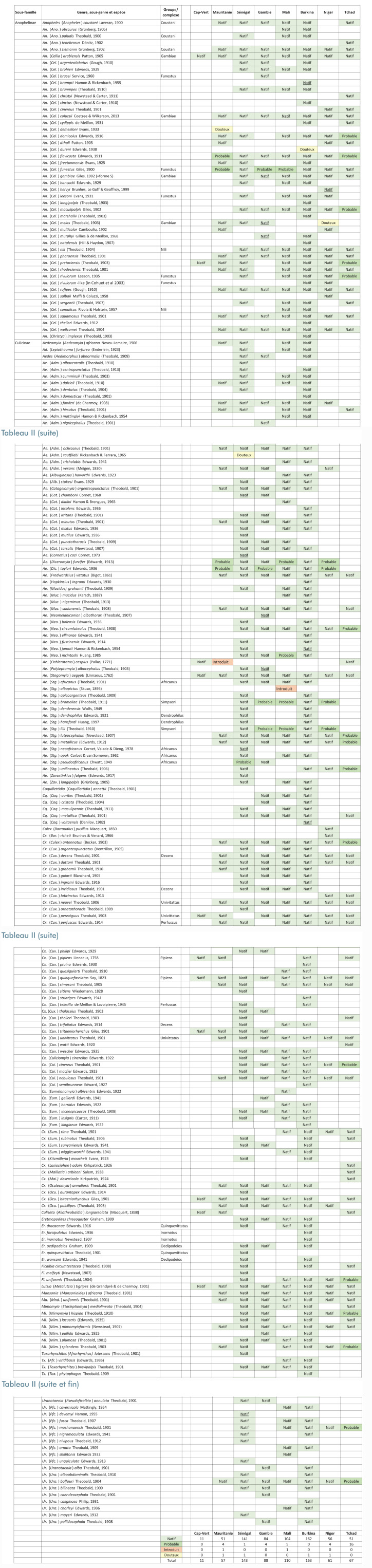

Les deux sous-familles de Culicidae, Anophelinae et Culicinae, sont représentées. Elles totalisent respectivement 48 et 168 espèces. Le nombre d'espèces d'Anophelinae par pays est toujours inférieur à celui des Culicinae, entre 18% (Cap-Vert et Sénégal) et 39% (Niger).

La sous-famille Anophelinae comprend le seul genre *Anopheles*, lui-même composé de 3 sous-genres, *Anopheles, Cellia* et *Christya*, représentés par 5, 42 et 1 espèces, respectivement. La sous-famille Culicinae comprend 12 genres: *Aedeomyia* (2 espèces), *Aedes* (62), *Coquillettidia* (6), *Culex* (54), *Culiseta* (1), *Eretmapodites* (7), *Ficalbia* (3), *Lutzia* (1), *Mansonia* (2), *Mimomyia* (7), *Toxorhynchites* (4) et *Uranotaenia* (19).

Ces 216 espèces représentent 6,0% (216/3581 [108]) de la richesse spécifique mondiale des Culicidae. L'absence d'*An.* (*Cel.*) *stephensi* Liston, 1901 dans la zone des huit pays ici considérés, est soulignée.

Les sous-espèces retenues sont au nombre de 24 (Tableau S9, à consulter sur le site de la Revue). Elles se répartissent parmi les espèces des genres *Anopheles* (7 sous-espèces), *Aedes* (6), *Culex* (5), *Eretmapodites* (2), *Mansonia* (1), *Toxorhynchites* (1) et *Uranotaenia* (2).

Parmi les espèces mentionnées au moins une fois dans la littérature, mais ici placées dans la catégorie ‘Absent', on note: *Ae. lineatopennis* au Burkina et au Mali (Tableau S1, S4, à consulter sur le site de la Revue), *An. maliensis* et *An. schwetzi* au Mali (Tableau S4, à consulter sur le site de la Revue), *Cx. ventrilloni* et *Ur. neireti* au Sénégal (Tableau S6, à consulter sur le site de la Revue).

Les pays hébergeant la plus grande richesse spécifique sont le Burkina (163 espèces dont 1 ‘Douteux'), le Sénégal (143 espèces dont 1 ‘Douteux') et le Mali (110 espèces); le pays avec la plus faible richesse est le Cap-Vert (11). Cette richesse est moindre au nord en climat hyper-aride et elle est supérieure au sud en climat sub-humide. Ainsi, la Mauritanie et le Niger, seuls pays continentaux sans zone sub-humide (Fig. 1) sont ceux avec la richesse spécifique la plus faible (57 dont 1 ‘Douteux', et 61 espèces dont 1 ‘Douteux', respectivement).

Le Tchad est le pays le moins bien inventorié. Deux arguments soutiennent cette conclusion: on estime à 16 le nombre d'espèces dont la présence y est ‘Probable' quoique non démontrée, ce nombre ne dépassant pas 5 pour chacun des sept autres pays (Tableau II); le Tchad et la Mauritanie sont les seuls pays de la zone d'étude où aucune espèce ou sous-espèce nouvelle pour la science n'a été décrite (Tableau III).

**Tableau III:** Nombre de localité-type pour les espèces et les sous-espèces culicidiennes décrites dans les huit pays de la zone d'étude Number of type-locality for the culicidian species and sub-species described in the eight countries of the study area

	Cap-Vert	Burkina Faso	Gambie	Mali	Mauritanie	Niger	Sénégal	Tchad	Total
Espèce	0	4	5	1	0	1	4	0	15
Sous-espèce	1	1	0	1	0	0	0	0	3
**Total**	**1**	**5**	**5**	**2**	**0**	**1**	**4**	**0**	**18**

**Figure 2:**
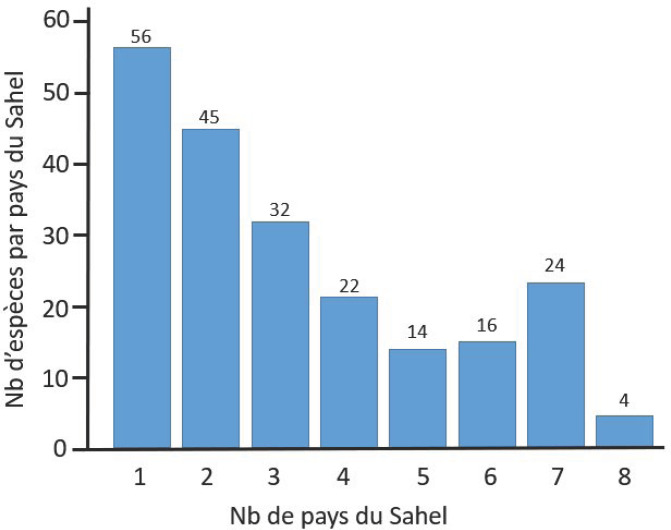
Nombres d'espèces de moustiques par pays. Ces nombres sont issus du Tab. 2 en excluant les catégories de présence ‘Douteux' (total = 213) Numbers of mosquito species per country. These numbers come from the Tab. 2 excluding the doubtful records (total = 213)

La Figure 2 montre le nombre d'espèces de moustiques présentes en fonction du nombre de pays de la zone sahélienne étudiée.

Cette distribution est globalement décroissante. Cinquante-six espèces sont présentes dans un seul pays, et seulement 4 sont présentes dans chacun des 8 pays. Ces dernières, *An. arabiensis, Ae. aegypti, Cx.* (*Cux.*) *quinquefasciatus* Say, 1823 et *Lutzia* (*Metalutzia*) *tigripes* (de Grandpré & de Charmoy, 1901), appartiennent à des genres différents et semblent présentes dans tous les pays africains, au sud du Sahara, Madagascar inclus. Les espèces présentes dans 7 pays sont au nombre de 24 (21 dans les 7 pays continentaux), avec 9 *Anopheles*, 3 *Aedes*, 8 *Culex*, 2 *Mansonia*, 1 *Mimomyia* et 1 *Uranotaenia*. Les espèces présentes dans un seul pays sont au nombre de 56, avec 12 *Anopheles*, 14 *Aedes*, 2 *Coquillettidia*, 15 *Culex*, 3 *Eretmapodites*, 1 *Ficalbia*, 2 *Toxorhynchites* et 7 *Uranotaenia* (Tableau S10, à consulter sur le site de la Revue).

Les espèces prédominantes dans l'écozone du paléarctique occidental sont peu nombreuses, telles que *Ae. vexans, Cx.* (*Lasiosiphon*) *adairi* Kirkpatrick, 1926, *Cx.* (*Maillotia*) *arbieeni* Salem, 1938, *Cx.* (*Mai.*) *deserticola* Kirkpatrick, 1924, *Cx. pipiens* et *Culiseta* (*Allotheobaldia*) *longiareolata* (Macquart, 1938). Les espèces à prédominance du paléarctique (occidental + oriental) et de la région indo-malaise sont encore moins nombreuses: *Ae. caspius, Cx.* (*Barraudius*) *pusillus* Macquart, 1850, *Cx. perexiguus, Ur.* (*Pseudoficalbia*) *unguiculata* Edwards, 1913. D'autres espèces ont une prédominance afro-tropicale, mais leur aire de répartition n'y est pas limitée et elles ont largement colonisé la sous-région méditerranéenne de l'éco-zone paléarctique: *An.* (*Cel.*) *pharoensis* Theobald 1901, *An. rufipes, An. rhodesiensis, Cx.* (*Cux.*) *antennatus* (Becker, 1903), *Cx.* (*Cux.*) *theileri* Theobald, 1903, *Cx.* (*Ocu.*) *poicilipes* (Theobald, 1903), et/ou débordant largement dans la région orientale: *An. dthali, An. multicolor, An. sergentii, Ae. sudanensis, Ae.* (*Fredwardsius*) *vittatus* (Bigot, 1864), *Cx.* (*Cux.*) *tritaeniorhynchus* Giles, 1901 et *Ma.* (*Mnd.*) *uniformis* (Theobald). Les autres espèces, soit une très large majorité, sont typiquement afro-tropicales. Une espèce introduite fait figure d'exception, *Ae. albopictus*, cantonnée au début du 20^e^ siècle au sud-est asiatique et aux îles du sud-ouest de l'océan Indien; elle s'est révélée envahissante et se retrouve maintenant sur tous les continents sauf l'Antarctique.

## Discussion

Dans cet inventaire des présences/absences, un point à considérer prioritairement est celui de l'hétérogénéité méthodologique. À l'évidence, il n'y a pas de concertation ou d'harmonisation méthodologique de l'échantillonnage entre tous les travaux qui ont servi au présent article; les enquêtes ont différé pour les enquêteurs, les dates, les moyens et les objectifs. Sans qu'il semble possible de le vérifier, les espèces anthropophiles sont, a priori, mieux inventoriées que les zoophiles ou zoophages, elles-mêmes mieux inventoriées que les moustiques dont on ignore tout des préférences trophiques. Il en va très probablement de même avec les espèces endophiles pour le comportement de repos, mieux inventoriées et donc plus aisées à collecter que les exophiles. La répartition des efforts de collecte entre les stades aériens (adultes femelles et/ou mâles) et aquatiques (larves et nymphes) est une autre source d'incertitude. Les approches morphologiques sont historiquement dominantes mais l'essor récent de nombreuses techniques moléculaires apporte de nouvelles connaissances. Il convient aussi de mentionner que les spécimens d'espèces rares, donc ultra-minoritaires dans les collectes, ont une forte propension à passer inaperçus dans les lots de spécimens d'espèces abondantes et bien connues. Tout cela découle du fait que les études ont surtout été réalisées dans un esprit « vecteurs » et les autres spécimens, à détermination plus difficile, sont alors sécurisés en collection pour être examinés plus tard, autorisant alors d'autres examens et analyses [9]. En revanche, la quantité des travaux réalisés, et la diversité des approches pratiquées, est de nature à réduire quelque peu cette hétérogénéité. De fait, les principaux résultats sont cohérents: plus faible nombre d'espèces dans le pays insulaire (Cap-Vert) et dans les pays les plus sahariens (Mauritanie, Niger); plus grand nombre d'espèces dans les pays présentant une zone sub-humide (Burkina, Sénégal, Mali); grand nombre d'espèces (56 espèces) distribuées dans un seul pays versus petit nombre d'espèces (4 espèces) présentes dans les 8 pays.

Concernant les 4 espèces, *An. arabiensis, Ae. aegypti, Cx. quinquefasciatus* et *Lt. tigripes*, présentes dans chacun des 8 pays, il est notable que les trois premières sont extrêmement liées à l'Homme, qui a probablement assuré la dissémination pantropicale d'*Ae. aegypti* et de *Cx. quinquefasciatus*. *Aedes aegypti* est un cas particulier dans la mesure où une population sauvage très peu liée à l'homme se maintient dans les zones boisées, assez isolée de la population domestique, pondant dans les trous d'arbre et les cosses de fruit, se nourrissant préférentiellement sur les animaux sauvages, et ne pénétrant pas dans les maisons [71]. Quant à *Lt. tigripes*, il semble difficile de relier sa vaste distribution aux traits particuliers de la biologie des larves (prédatrices d'autres larves de moustiques) ou des femelles adultes (principalement ornithophiles et exophiles); il est possible que la relative grande taille des larves et adultes ait attiré l'oeil du collecteur.

Les 25 espèces présentes dans les 7 pays continentaux de cette zone d'étude appartiennent à 6 genres majoritairement représentés par les *Anopheles* (38%) et les *Culex* (33%). La quasi-totalité est couramment retrouvée dans les enquêtes entomologiques qui ne sont pas orientées vers la connaissance de la biodiversité, mais le plus souvent viennent en complément de recherches sur les études de la transmission vectorielle des pathogènes humains et/ou vétérinaires.

L'absence actuelle d'*An. stephensi* est importante car cette espèce envahissante est d'ores et déjà présente à l'ouest du golfe Persique. Elle s'est d'abord implantée en Arabie saoudite, puis à Djibouti en 2012, puis en Ethiopie, puis au Soudan [127, 130], si bien que l'introduction de ce vecteur majeur de *Plasmodium* humains est redoutée dans les huit pays ici étudiés [130].

Dans quelles mesures l'écologie des stades aquatiques gouverne-t-elle la distribution des espèces? Les 28 espèces culicidiennes les mieux représentées (présentes dans au moins 7 pays de la zone d'étude) se répartissent dans les genres culicidiens suivants: 10 *Anopheles*, 9 *Culex*, 4 *Aedes*, 2 *Mansonia*, 1 *Mimomyia*, 1 *Lutzia* et 1 *Uranotaenia* (Tableau S10, à consulter sur le site de la Revue). L'écologie larvaire de ces espèces est principalement inféodée à des gîtes d'eau douce; 13 espèces ne peuvent survivre que dans une eau sans sels minéraux, comme c'est le cas pour *An. funestus, An.* (*Cel.*) *squamosus* Theobald, 1901 et *An. wellcomei*. Aucune espèce n'est véritablement halophile, mais 3 (*Ae. vittatus, Cx. antennatus, Cx. perexiguus*) peuvent occasionnellement supporter une très faible teneur en sel et 2 *Anopheles* (*An. pharoensis* et *An. ziemanni*). peuvent supporter une légère salinité. Parmi les 28 espèces, 11 sont couramment collectées dans des gîtes larvaires permanents, de grande taille et associés à une abondante végétation aquatique ou herbeuse: *An. funestus, An. rufipes* (de la sous-espèce *broussesi*), *An. pharoensis, An. squamosus, An. ziemanni, An. wellcomei, Cx. antennatus, Ma. africana, Ma. uniformis, Mi.* (*Mim.*) *Mimomyia* formis (Newstead, 1907) et *Ur.* (Ura.) *balfouri* Theobald, 1904. La sous-espèce *broussesi* d'*An. rufipes* est toujours associée à une abondante végétation et fréquemment rencontrée dans les puits creusés dans les oueds; *Cx. bitaeniorhynchus* est collecté dans l'eau claire des réservoirs parmi des algues vertes filamenteuses. Les espèces potentiellement les mieux représentées dans les périmètres rizicoles irrigués sont au nombre de 9, dont 7 *Anopheles* (*An. arabiensis, An. coluzzii, An. funestus, An. pharoensis, An. rufipes, An. squamosus* et *An. ziemanni*), 1 *Culex* (*Cx. antennatus*) et 1 *Uranotaenia* (*Ur. balfouri*). Les espèces de trou d'arbre sont principalement des *Aedes* du sous-genre *Stegomyia* (*Ae. aegypti, Ae.* (*Stg.*) *luteocephalus* (Newstead, 1907), *Ae.* (*Stg.*) *metallicus* (Edwards, 1912) et 1 *Culex* du sous-genre *Culiciomyia* (*Cx. nebulosus*); ces 4 espèces peuvent également être capturées dans des gîtes domestiques anthropiques, cohabitant parfois avec *Cx. quinquefasciatus* et *Lt. tigripes*. Enfin, *Cx. quinquefasciatus* et *Cx.* (*Cux.*) *duttoni* Theobald, 1901 sont associés aux gîtes aquatiques d'origine humaine chargés en matière organique comme les latrines pour la première et l'eau boueuse des emprunts de terre pour la seconde. Pour conclure sur l'écologie des stades aquatiques, il n'y a pas de type de gîte aquatique réellement dominant parmi ces espèces les mieux distribuées. Certaines espèces ont une écologie larvaire liée aux gîtes naturels permanents ou sub-permanents, comme les bords d'étang ou de marigot, les mares, d'autres ont une écologie directement liée à l'activité humaine.

Au final, l'impression générale qui se dégage de cette synthèse est que la connaissance de la distribution des espèces de moustiques dans ces pays sahéliens est perfectible. À l'issue du présent travail de recensement, 3,0% des mentions du Tableau II restent incertaines (18 ‘Probable' + 3 ‘Douteux' / un total de 700 mentions). Plusieurs raisons peuvent être invoquées pour expliquer cette situation:

- des identifications sont impossibles, car de nombreux stades restent inconnus. Parmi les 216 espèces retenues dans la zone d'étude, 28 (soit 13%) sont dans ce cas (7 espèces pour lesquelles le mâle reste non décrit, 6 pour la femelle, et 25 pour la larve) (Tableau S11, à consulter sur le site de la Revue). Les enquêtes entomologiques consacrées aux vecteurs (biologie, agressivité pour les sujets humains, etc.) ne considèrent guère les mâles;
- la détermination spécifique d'un spécimen est souvent difficile, ce qui soulève la question de la formation en entomologie et de l'intérêt des enquêtes au niveau régional. Il faut déplorer le manque d'outils d'identifications (type clé dichotomique ou logiciel d'identification) valables pour tous les moustiques de la zone sahélienne ici considérée. Les méthodes disponibles concernent la totalité de l'Afrique sub-saharienne et donc un nombre excessif d'espèces [20, 56, 77, 126] ou bien sont anciennes et incomplètes, en particulier pour les *Aedes* et les *Culex* [46, 79];
- les enquêtes permettent souvent de récolter des milliers de moustiques et la très grande majorité des spécimens identifiés n'est pas conservée: une erreur éventuelle d'identification peut longtemps perdurer dans la littérature avant qu'elle soit – ou non – corrigée. La situation s'améliore grâce aux méthodes moléculaires qui complètent la traditionnelle identification morphologique en apportant un réel supplément de crédibilité au diagnostic d'espèce [9]. La connaissance du complexe Gambiae et du groupe Funestus ainsi que des espèces morphologiquement proches du groupe Univittatus (*Cx. univittatus, Cx. neavei, Cx. perexiguus*) en a déjà bénéficié [103];
- les prospections se focalisent surtout sur les espèces d'intérêt médical ou vétérinaire, au premier rang desquelles les espèces anthropophiles vectrices, et ne concernent pas les espèces rares ou associées à la faune sauvage et/ou à des phytotelmes;
- l'absence d'indications géographiques ne permet pas toujours de retrouver la localisation précise de la collecte du spécimen (lieu mal orthographié ou non référencé sur les cartes, déplacement du village, homonymie de localités). L'avènement des relevés GPS est une vraie amélioration;
- les problèmes de sécurité des personnes deviennent considérables dans certaines parties du Sahel et rendent durablement inenvisageables les prospections dans ces zones.

## Conclusion

La présente étude fait la synthèse des connaissances sur la distribution de 216 espèces de moustiques présentes dans la zone couverte par 8 pays sahéliens d'Afrique.

Les espèces les mieux documentées sont les Anophelinae, les moustiques vecteurs ou les moustiques agressifs pour l'Homme; à l'inverse, les espèces les moins bien documentées sont des Culicinae, zoophiles ou dénuées d'intérêt médical ou vétérinaire connu.

Un lien existe entre les informations disponibles (quantitativement et qualitativement) et la présence de structures universitaires. Il apparaît aussi que le niveau de recherche est variable selon les pays et soulève la question du manque d'entomologistes et d'enquêtes standardisées. Cette synthèse, largement placée sous le signe de la richesse spécifique comme marqueur de biodiversité, devrait servir aux recherches futures portant sur la destruction, la fragmentation et la préservation des milieux naturels, le changement climatique, et l'émergence de nouveaux pathogènes à transmission vectorielle. D'ores et déjà, elle peut être utile dans les domaines de la lutte antivectorielle et de la santé publique.

## Remerciements

Tous les auteurs relèvent de laboratoires membres du réseau entomologie de MediLabSecure, projet européen visant à prévenir les maladies dues à des arbovirus et à renforcer les compétences dans les régions de la mer Méditerranée et du Sahel. MediLabSecure <www.medilabsecure.com > est un projet financé par la Commission européenne (EU DG DEVCO: IFS/2018/402-247).

Richard C. Wilkerson et Yvonne-Marie Linton, du Department of Entomology, National Museum of Natural History, Smithsonian Institution, Washington DC, sont remerciés pour avoir partagé des renseignements relatifs à la base Walter Reed Biosystematics Unit.

## Avertissement

Le contenu de cet article est de la seule responsabilité des auteurs et ne reflète pas nécessairement les points de vue de l'Union européenne.

## Conflits D'intérêts

Les auteurs ne déclarent aucun conflit d'intérêts.
